# The impact of fluconazole use on the fungal and bacterial microbiomes in recurrent Vulvovaginal Candidiasis (RVVC): a pilot study of vaginal and gastrointestinal site interplay

**DOI:** 10.1007/s10096-024-04999-1

**Published:** 2024-11-26

**Authors:** Moira Bradfield Strydom, Tiffanie M. Nelson, Sohil Khan, Ramesh L. Walpola, Robert S. Ware, Evelin Tiralongo

**Affiliations:** 1 School of Pharmacy and Medical Sciences, Griffith University, Gold Coast, QLD Australia; 2 Biostatistics Unit, Griffith Health, Griffith University, Gold Coast, QLD Australia; 3 School of Health Sciences, Faculty of Medicine and Health, UNSW Sydney, Sydney, NSW Australia

**Keywords:** Recurrent vulvovaginal candidiasis, Vaginal microbiome, Fluconazole, Maintenance therapy, Mycobiome

## Abstract

**Purpose:**

Recurrent Vulvovaginal Candidiasis (RVVC) is a problematic clinical condition for which fluconazole treatment is commonly prescribed. This study investigated the interkingdom vaginal and gastrointestinal microbiomes of RVVC patients who use fluconazole intermittently or as longer-term maintenance therapy for symptom management and compared them to healthy controls.

**Methods:**

Vaginal swabs and fecal samples were collected. A novel interkingdom analysis was performed using 16 S rRNA and ITS1 gene sequencing to compare the diversity and taxonomic composition of vaginal microbiome (VMB) and gastrointestinal microbiome (GIMB).

**Results:**

Twenty-seven women participated: 10 intermittent users and healthy controls and 7 maintenance therapy. The study revealed that microbiomes of fluconazole users do not differ in diversity metrics from healthy controls. RVVC patients using intermittent fluconazole displayed a higher abundance of vaginal *C. albicans* than healthy controls. *Candida* species pairings were not commonly observed between sites in individuals and, as such a fecal reservoir is unlikely to be implicated in recurrent symptomatology. In many of the RVVC non-*Candida* fungal spp. were identified in the vaginal microbiome. Users of fluconazole displayed elevations of the CST-I (Community State Type 1) associated bacterium *L. crispatus.* All participants displaying vaginal *Candida* spp. belonged to either bacterial CST-I or CST-III (Community State Type 3- *L. iners* associated).

**Conclusion:**

To our knowledge, this is the first study to compare the interkingdom VMB-GIMB of women with RVVC using oral fluconazole. As fluconazole users in this study represent a typical RVVC population, trends observed in microbial abundance require further analysis to establish fluconazole’s long-term microbiome safety. Examining the microbiome at both sites adds to the current understanding of microbial associated with the condition.

**Supplementary Information:**

The online version contains supplementary material available at 10.1007/s10096-024-04999-1.

## Introduction

As our understanding of the human microbiome increases, so too does our understanding of the role specific microbes have on whole organism microbiome function. Imbalances driven by individual species, such as *Candida albicans*, can disrupt homeostasis with adverse consequences to human health [1]. One example is Recurrent Vulvovaginal Candidiasis (RVVC), which affects approximately 10% of the female adult population with impacts on both their physical and emotional well-being [1, 2]. Several *Candida* species have been implicated in RVVC, with symptoms including pruritis, vulvovaginal inflammation, dyspareunia, external dysuria, and abnormal vaginal discharge [3]. Some patients will manage their RVVC symptoms with intermittent fluconazole therapy consisting of sporadic administration of the drug as needed but for many, this type of episodic treatment is insufficient to ensure a long-term cure [4, 5]. The alternative management for prolonged symptom remission is ongoing daily or weekly fluconazole maintenance therapy for up to six months at varying doses [3, 6]. Whilst symptom control is largely achieved with maintenance therapy, effects are not sustained post therapy. Up to 57% of patients relapse within 3–6 months of ceasing treatment [4, 5]. The significant relapse rate and lack of safe alternate interventions has led to the clinical practice of indefinite fluconazole prescription to provide ongoing symptomatic relief from RVVC [1]. However, although adverse effects from taking fluconazole are uncommon [7], extended, and potentially indefinite, treatment may have undefined repercussions on the user’s vaginal microbiome (VMB) and gastrointestinal (GIMB) microbiome [1, 2]. Little is known about the microbiome impacts of oral fluconazole outside of its ability to reduce *Candida* species but commonly used drugs, such as e.g. fluconazole, can cause significant and lasting changes in the metabolic function and taxonomy of an individual’s microbiome with unclear consequences [8].

There are inconsistencies in understanding which microbiota patterns contribute to RVVC persistence [9–12]. Evidence exists for specific *Lactobacillus* species-level dominance; *Candida* and *Lactobacillus* relationships; *Candida* pathogenicity in relation to vaginal pH levels; and the potential transfer of microorganisms between the gastrointestinal tract (GIT) and vagina [12, 13].

Mucosal barriers like those found in the GIT and the vagina are acknowledged as a hub for interkingdom (fungal and bacterial) communication and communication between microbiota and host [14]. There is very little known about the interkingdom microbiome (bacterial and fungal microbiome combined) in RVVC individuals, such as the role of non-*Candida* fungal species in bacterial interactions and the overall fungal burden symptomatology and treatment response. The GIMB is of interest in RVVC as not only does oral fluconazole interact directly with the interkingdom microbiome in the gut, the GIT is often purported to be a source of opportunistic *Candida* spp. translocation to the vagina [15]. Only one study has examined GIT *Candida* carriage in RVVC during fluconazole therapy, finding a link between a decreased response to digressive dosing fluconazole maintenance therapy and multi-site carriage [16]. Digressive dosage is a maintenance therapy that reduces dosage over time to find the lowest dosage providing symptomatic suppression in a weekly, fortnightly or monthly dosage [17]. No studies have been conducted which report on the *Candida* spp. co-carriage and pairing relationship in the GIMB and VMB of RVVC patients on varying fluconazole regimes. Impacts from intermittent and maintenance fluconazole therapy on the GIMB and VMB in these individuals have also not been defined. A comparison to the microbiomes of healthy controls is also lacking.

In this exploratory study we investigated the interkingdom VMB and GIMB of people with RVVC on either intermittent (IF) or maintenance (MF) fluconazole therapy to understand possible effects on microbiome characteristics when compared to healthy controls (HC). A novel merged interkingdom analysis was utilised to provide insight via 16 S rRNA and ITS1 gene sequencing comparing the diversity and taxonomic composition of GIMB and VMB.

## Materials and methods

### Study participants

Healthy women with RVVC aged between 18 and 50 years were recruited Australia-wide between September 2021 and July 2022 *via* social media, pharmacies, general and specialist medical practices and the university. Eligible participants, including those with RVVC utilising fluconazole as their primary treatment strategy, were identified *via* an initial online questionnaire and subsequent phone screening. Exclusion criteria were: use of an intrauterine device (IUD), autoimmune disease (except medicated Hashimoto’s disease), sexually transmitted infections (except latent herpes simplex virus 1 and 2), or other vulvar or vaginal conditions, pelvic inflammatory disease, active human papilloma virus (HPV), GIT disorders, and cancer. The study also excluded patients taking medications or treatments that affected GIT pH, hormones (except combined oral contraceptives and thyroxine) as well as immunosuppressants, chemotherapy, anti-inflammatories. Those using antibiotics (including herbs and nutrients with antimicrobial effects), or probiotics (orally or vaginally) were ineligible except for those willing and able to cease medications at least 2 weeks before study entry. Participants in the intermittent fluconazole (IF) group were excluded if they had used fluconazole in the 14 days prior to sample day. Potential participants who had received a vaccination two weeks before sample collection were asked to delay study participation by a month or were excluded. Any participant that performed douching, was pregnant or menopaused was also excluded.

Participants were enrolled in one of three groups: non-RVVC healthy control (HC), RVVC patients with intermittent fluconazole use (IF) and, RVVC patients with maintenance fluconazole use (MF) (Table S1). Intermittent fluconazole treatment was defined as use of fluconazole as a single dose three times or more in the preceding 12 months, and MF users used fluconazole at least once per week (dosage could vary from 50 mg-1400 mg a week) or in a repetitive prescribed fortnightly dosing pattern for at least a month prior to study entry. All participants were required to be symptom-free on enrollment and sample collection day.

### Ethics approval and consent

Approval for the study was obtained through the Griffith University Human Research Ethics Committee (Ref No.2021/674). Participants provided written consent for sample and data collection and subsequent analysis.

### Sample collection

Participants attended a teleconference prior to providing any samples. The purpose of the teleconference was to collect baseline data *via* a researcher-led study entry questionnaire and to ensure confidence in the sampling procedure. Baseline data included demographics, medication use, birth and early life feeding practices and menstrual cycle data.

Vaginal samples were self-collected utilising *FloQ swab*^*®*^(Copan) in the mid-luteal phase of the participant’s menstrual cycle or calculated cycle equivalent. A 48-hour abstinence period was required, and no lotion, creams, or emollients could be applied to the vulvovaginal area on the day of swab collection. Swabs were collected two hours away from bathing and not when menstruating. Swab one was reserved for bacterial sequencing and swab two for fungal sequencing. A third sterile swab was used by participants to determine vaginal pH using a PEHANNON^®^ pH strip (Marchery-Nagel). On the same day participants also collected a fecal sample using an OMNIGENE GUT^®^ kit (DNAGenoTek). All remote samples were immediately sent back to Griffith University’s Clinical Trial Unit (CTU) *via* express next-day post/courier or returned in person (0–24 h until receipt of sample). Samples were stored at -80 °C at the CTU until shipped to the Australian Genome Research Facility (AGRF, Adelaide, SA, Australia).

On sampling day participants were required to complete a questionnaire which included questions about their current menstrual cycle, menstrual hygiene practices and sexual interaction in the two weeks preceding sample collection (Table S2- S3). Participants also recorded their vaginal pH and completed a symptom score using the Sobel score [4].

### DNA extraction and sequencing

Sample processing and analysis was conducted by the AGRF in accordance with their protocols. Briefly, Genomic DNA was extracted from swab 1 and 2 using the DNeasy PowerSoil Pro Kit (Qiagen, Germany). Extracted genomic DNA from bacteria and fungi were used as a template in PCR reactions using the Platinum SuperFi II mastermix (Life Technologies, Australia) to amplify regions from the 16 S rRNA gene (V3-V4) or ITS region (ITS1) using primers to target each region (Table 1), respectively. Thermal cycling was performed in an Applied Biosystem 384 Veriti Thermal Cycler. PCR amplicon generation was performed using separate reaction tubes for individual primers sets in. Thermal cycling conditions after an initial reaction of 98 °C for 30 s, were as follows: 30 cycles at 98 °C for 10 s, 60 °C for 10 s and 72 °C for 30 s with a final extension step of 72 °C for 5 min. The resulting PCR amplicons were sequenced using an Illumina MiSeq instrument (San Diego, CA, USA) with a 600-cycle kit (2 × 300 base pairs paired-end). Sequence data are available on the National Center for Biotechnology Information (NCBI) Sequence Read Archive under BioProject accession number SUB14294064.

**Table 1:** Primer sets used for targeting bacterial and fungal regions for amplification

Region	Primer	Primer Sequence	Amplicon size (bp)	Reference
Bacterial V3-V4 region of the 16 S rRNA gene	341 F	CCTAYGGGRBGCASCAG	300	[18]
	806R	GGACTACNNGGGTATCTAAT		
Fungal ITS1 internal transcribed spacer region (ITS) region	ITS1	CTTGGTCATTTAGAGGAAGTAA	300	[19]
	ITS2	GCTGCGTTCTTCATCGATGC		

### Bioinformatics

Raw fungal and bacterial sequences were converted into separate Amplicon Sequence Variant (ASV) abundance table using the QIIME2 (Quantitative Insights Into Microbial Ecology) version 2019.7 suite of tools [20]. Demultiplexed sequence read counts from all vaginal and fecal samples for the 16 S region ranged between 11,598 and 252,723 and 0 to 264,957 for the ITS region (Table S4). Participants with one or less sequence read counts were removed from downstream analyses. The demultiplexed raw reads were primer trimmed and quality filtered using the cutadapt plugin, followed by denoising with DADA2 [21]. Taxonomy was assigned to ASVs using the q2-feature‐ classifier [22]. Further identification of taxa was obtained through BLAST (Basic Local Alignment Search Tool) via the National Center for Biotechnology Information interface (blast.ncbi.nlm.nih.gov/Blast.cgi).

### Statistical analysis

The association between participant characteristics and fluconazole user groups was investigated using Fisher’s Exact Test for categorical data and analysis of variance for continuous variables. ITS and 16 S region data were merged into a single novel dataset for each site, i.e., GIT and vagina, to investigate novel interkingdom dynamics. The merged dataset consisted of 524 taxa with 399 assigned to the kingdom Bacteria, 123 assigned to the kingdom Fungi and two unassigned at the level of kingdom. Data were pruned to remove representatives not classified at the level of kingdom (*n* = 3) taxa assigned to the kingdom Archaea (*n* = 6), and additionally taxa classified to mitochondria (*n* = 2) at the taxonomic level of family. To identify specific characteristics of the microbiome community, downstream analyses from the vagina and GIT were treated separately for each region (16 S and ITS) where required. Data treatments are described as merged and unmerged when discussed to distinguish between the different treatment methods. ASV filtering and pruning was conducted with the *phyloseq* package version 1.42.0 [23] in the R statistical program version 4.2.1 Vienna, Austria [24].

Rarefaction curves (Figure S1) did not identify significant loss for samples from the 16S region for both the vaginal and GIT sites. Curves indicate the low abundances from the ITS region at both sites, yet lower counts also showed a plateau of the curve. To account for differences in sampling depth in the merged dataset and in the 16S specific region for the vagina and GIT, data were rarefied to the lowest depth (96,423 in rarefied vaginal dataset, 13,776 in the rarefied GIT dataset). Alpha diversity metrics including observed, Shannon’s [25] and Chao [26] were estimated based on ASVs and visualized with the *phyloseq* package version 1.42.0 in R [24]. Kruskal-Wallis one-way analysis of variance was used for comparison between groups of alpha diversity indices. To identify beta diversity across samples, datasets for each sampling site, (‘unmerged’), GIT and vagina, were visualised and tested for significant differences separately. Differences in the microbiome as measured by the Bray Curtis dissimilarity matrices [27] were visualised on a Principal Coordinates Analysis plot and tested for differences by conducting a permutational multivariate analysis of variance (PERMANOVA) using the command “adonis2” in the package *vegan* version 2.6.4 in R version 4.2.1 [24] with the grouping factor ’treatment’. Results for all statistical tests were considered significant where p-values < 0.05. An individuals VMB was categorised into one of five community state types (CST’s) as determined by the dominant *Lactobacillus* species in the microbial community [28]. The CST of study participants was determined by comparing the relative abundance (unmerged) of *Lactobacillus* spp. identified in the vagina of each participant (Figure S2). Where any one *Lactobacillus* species displayed a dominant relative abundance [28, 29] it was categorised as follows: *L. crispatus*, CST-I; *L. gasseri*, CST-II; *L. iners*, CST III; *L. jensenii*, CST-V, and; no dominant lactobacilli, CST-IV.

### Sample size

Limited vaginal microbiome studies evaluating the RVVC microbiome are published featuring sample sizes of 40 participants on average. These studies have evaluated differences to either fungal or bacterial diversity at single and multiple timepoints, however, combined studies with whole microbiome analysis do not exist.

To explore the impacts of fluconazole treatment on the fungal and bacterial RVVC microbiome, recruitment was aimed at 40 participants across the three groups (15 in each of the IF and MF groups, 10 in HC) to show statistical differences in the Shannon diversity. A formal sample size calculation was not undertaken due to the exploratory nature of the study. To measure between-group change in overall Shannon diversity values derived from Bradford et al. (2017) were used, who demonstrated between-group differences in bacterial microbiome in a VVC population [30].

## Results

### Participants and basic characteristics

Twenty-seven participants (68% of planned enrolments) were allocated to one of three groups: control (HC, number of participants, *n* = 10), intermittent fluconazole user (IF, *n* = 10) and maintenance fluconazole user (MF, *n* = 7). Participant baseline characteristics are displayed in Table 2. The average age of participants was 31 (range: 18 to 41) years. No significant differences in ethnicity, menstrual cycle length, sexual activity, medication use or pregnancy history were observed between groups (Table 2, S2-S3, all *p* > 0.05). The average measure of vaginal pH was 5.0 +/- 0.4 with no significant differences observed between groups (Table S3, *p* = 0.49). Participants in the IF treatment group primarily took 150 mg as a single dose as required for symptomatic episodes (*n* = 9), while one participant required 50 mg three times in a week for their symptomatic recurrence (Table S1). Participants in the MF treatment group mostly took 50 mg of fluconazole daily (*n* = 4, Table S1). All participants in the MF group had used fluconazole in the four days leading up to their sample collection.

**Table 2:** Participant characteristics of the healthy control (HC), intermittent fluconazole (IF) and maintenance fluconazole (MF) groups

Variable		HC (*n* = 10)	IF (*n* = 10)	MF (*n* = 7)	*P*- Value
Age at enrolment years		32.1 ± 7.3	29.9 ± 6.2	29.4 ± 6.8	0.67
Ethnicity	*White* *Asian* *Black* *Hispanic*	7(70%) 2(20%) 0(0%) 1(10%)	8(80%) 1(10%) 1(10%) 0(0%)	7(100%) 0(0%) 0(0%) 0(0%)	0.77
Menstrual cycle length (days)		29.4 ± 5.0	28.8 ± 1.4	27.4 ± 1.6	0.48
Sexually active		9(90%)	8(80%)	6(85%)	1.00
Sexual activity type	*Not sexually active* *Unprotected sex* *Unprotected-oral contraceptive pill* *Unprotected-rhythm* *Unprotected-withdrawal* *Unprotected-diaphragm* *Barrier/Condom/Dam*	1(10.0%) 5(50.0%) 0(0.0%) 1(10.0%) 0(0.0%) 0(0.0%) 3(30.0%)	2(20.0%) 2(20.0%) 1(10.0%) 1(10.0%) 1(10.0%) 1(10.0%) 2(20.0%)	1(14.3%) 0(0.0%) 1(14.3%) 3(42.9%) 1(14.3%) 0(0.0%) 1(14.3%)	0.23
Medication	*OCP* *Antidepressant* *Beta-blocker* *Nutrient supplements (Mg*,* B12*,* Fe)* *Dexamphetamine sulphate* *Fluconazole - IF* ( use in last 14 days) *Fluconazole - MF* (use in last 4 days)	1(10.0%) 0(0.0%) 1(10.0%) 0(0.0%) 0(0.0%) 0(0.0%) 0(0.0%)	1(10.0%) 1(10.0%) 0(0.0%) 1(10.0%) 0(0.0%) 10(100.0%) 0(0.0%)	1(14.3%) 1(14.3%) 0(0.0%) 1(14.3%) 1(14.3%) 0(0.0%) 7(100.0%)	0.62
Pregnancy history	*Prior pregnancy* *Never pregnant*	3(30.0%) 7(70.0%)	2(20.0%) 8(80.0%)	4(57.1%) 3(42.9%)	0.27
Own birth type	*Vaginal Delivery* *Caesarean delivery*	9(90.0%) 1(10.0%)	10(100.0%) 0(0.0%)	6(85.7%) 1(14.3%)	0.72
Own early life feeding	*Breast/Chest fed* *Bottle fed* *Mixed fed* *Unsure*	7(70.0%) 1(10.0%) 1(10.0%) 1(10.0%)	10(100%) 0(0.0%) 0(0.0%) 0(0.0%)	4(57.1%) 0(0.0%) 3(42.9%) 0(0.0%)	0.05

Continuous data are expressed as mean and SD (standard deviation), and categorical data as n/N (%) (number of participants in grouping, [n] over total number of participants [N]). Significant differences were tested using Fisher’s Exact Test for categorical data and ANOVA for continuous variables.

### Characterisation of the interkingdom vaginal and gastrointestinal microbiomes

**Fig. 1:**
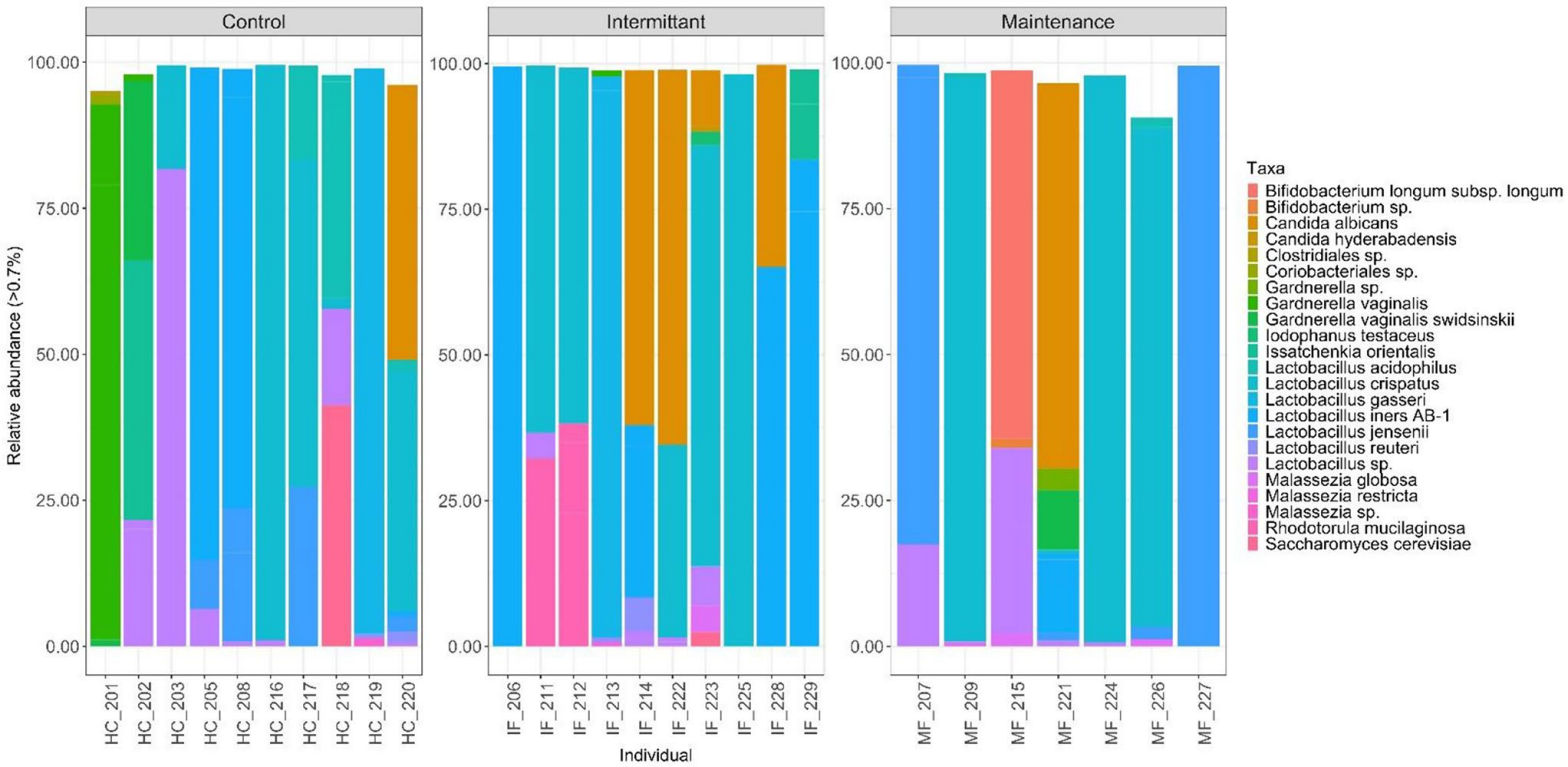
Relative abundance bacterial and fungal species in the vagina of participants. Bar plot shows the relative abundance of amplicon sequence variants (ASVs) with a relative abundance greater than 0.5% found in the vagina of the 27 participants, grouped by three treatment cohorts: healthy control (HC), RVVC patient with intermittent fluconazole use (IF) and RVVC patient with maintenance fluconazole use (MF). To account for differences in sampling depth between samples, vaginal data were rarefied prior to conversion to relative abundance. Colours indicate the summed relative abundance at the level of species from an individual vaginal swab sample

Following quality filtering the dataset generated from 27 participant samples yielded 7,451,272 sequence reads. Sequence read depths varied considerably between ITS and 16 S regions. Fewer reads were identified from the ITS region compared to the 16 S region despite the use of multiple swabs and adequate DNA concentrations. This reduced sequencing depth was more pronounced in the vaginal ITS dataset, where 19 of the 27 participants displayed a read count greater than 260 and qualified them for downstream analyses. All sequence reads, were classified into 522 taxa from the vagina and 3,629 taxa from the GIT. The interkingdom VMB consisted of taxa from eleven phyla from the Fungi and Bacteria dominated by the Firmicutes (average relative abundance = 74.6%), Ascomycota (14.1%), Actinobacteria (8.2%) and Basidiomycota (3.1%) (Fig. 1, Table S5). The VMB was represented by more than 80 genera with dominance from *Lactobacillus* (74.4%), *Candida*, (10.4%) and *Gardnerella* (5.0%) (Fig. 1, Table S5). A *Lactobacillus*-dominated environment was observed ranging from 0.02 to 98.5% relative abundance in any one participant. Eleven different *Lactobacillus* species were identified and numerous distinct ASVs were also identified, although classified only to the level of *Lactobacillus* (data not shown). The most common *Lactobacillus* species observed were *L. crispatus*,* L. gasseri*,* L. iners L. iners* A-B-1 and *L. jenseni* (Fig. 1). Other dominant taxa (with > 5% relative abundance in any one participant) belonged to seven different species: *Candida albicans*,* Gardnerella vaginalis*,* G. swidsinskii*,* Rhodotorula mucilaginosa*,* Bifidobacterium Longum subspo. Longum*,* Issatchenkia orientalis*,* Saccharomyces cerevisiae*,* and Malassezia globosa* (Fig. 1, Table S5).

The VMB profiles of the total cohort were clustered into five CST’s (Table 3). The most prevalent CST in participants was the *L. crispatus* dominated CST-I (40.7% of samples) followed by CST-III *L. iners* (25.9%) and then CST-IV non- lactobacillus dominated (11.1%). Participants with CST-II *L. gasseri*, and CST-V *L. jensenii* were both present in 7.4% of samples, while another 7.4% of CST’s were *Lactobacillus-*dominated by an unassigned species.

The GIMB of participants consisted of taxa from 12 phyla with dominance by the Firmicutes and Bacteroidetes and representation from the Actinomycetota, specifically in the genus, *Actinomyces* (Table S6). Dominant genera included *Prevotella* (15.4%), *Bacteriodes* (11.7%) and *Blautia* (9.2%).

**Table 3:** CST: Community state type frequency

Community State Type (CST)	Maintenance fluconazole (*n* = 7)	Healthy Control (*n* = 10)	Intermittent fluconazole (*n* = 10)
CST-I, *L. crispatus*	3	3	5
CST-II, *L. gasseri*	0	1	1
CST-III, *L. iners*	1	2	4
CST-IV, non*-Lactobacillus-*dominated	1 (C3-*Bifidobacteria* spp.)	2 (A-*G. vaginalis)*	0
CST-V, *L. jensenii*	2	0	0
Unassigned *Lactobacillus* spp.-dominated	0	2	0

### Differences in microbiome richness and diversity between treatment groups

**Fig. 2:**
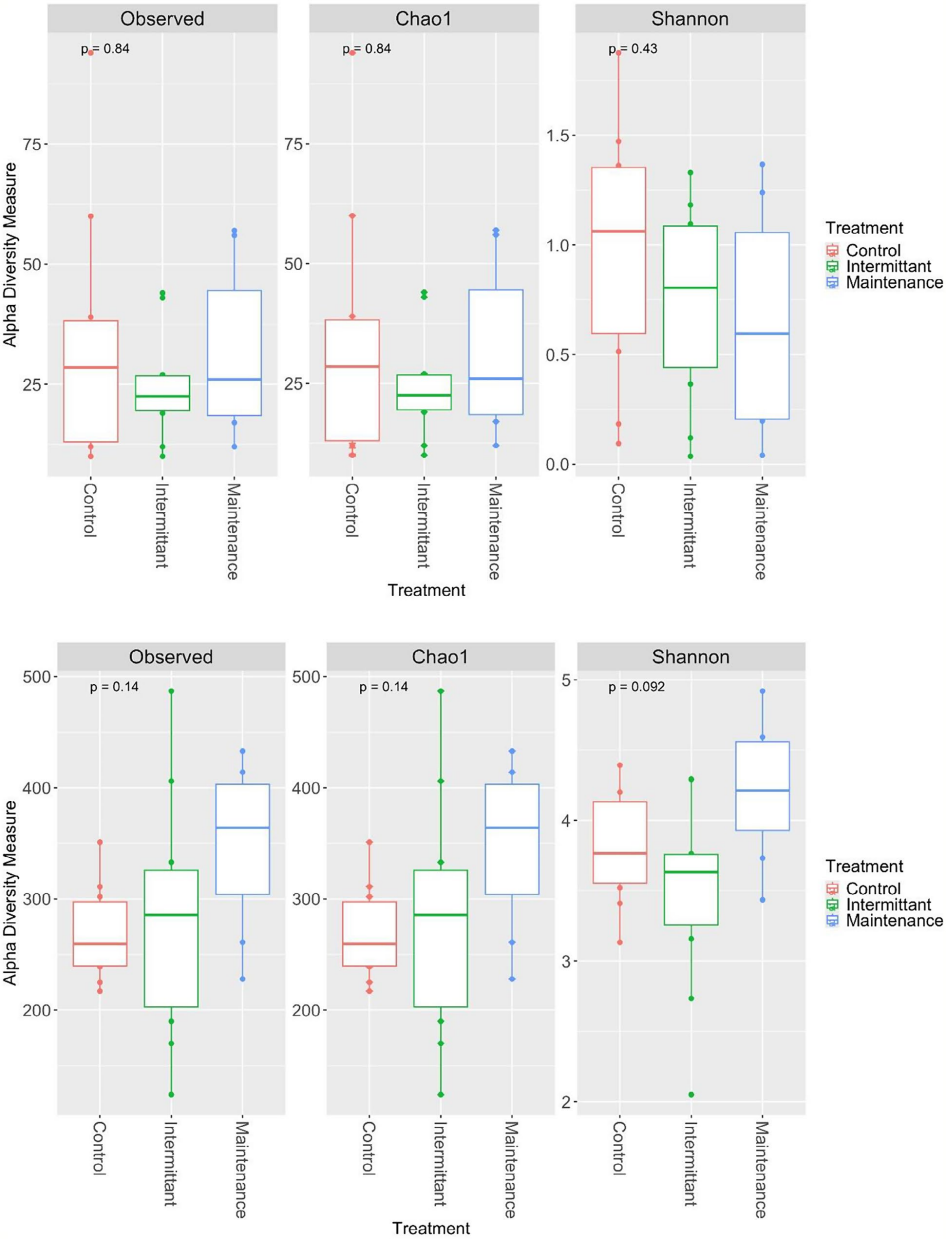
Alpha Diversity of fungal and bacterial microbiome in vagina and GIT locations with treatment for RVVC. Alpha diversity metrics showing observed number of amplicon sequence variants, Shannon and Chao diversity metrics of vagina (top) and GIT (bottom) in treatment groups indicating testing of the mean between groups with Kruskal-Wallis test

The Shannon index and Chao [25, 26] are methods of alpha diversity that are commonly used to characterise the richness and evenness of the microbial taxa present in a sample. Comparatively, beta diversity is a measure that indicates the similarity and differences in composition between samples. Overall, GIT samples displayed a far greater observed number of taxa and other alpha diversity metrics when compared to vaginal samples (Figure S2, Kruskal-Wallis, p-value = < 0.05). However, there were no differences in the observed number of taxa, Shannon’s or Chao diversity metrics between groups within the GIT or vagina (Fig. 2, p-value > 0.05). Vaginal samples displayed a relatively consistent trend in alpha diversity metrics across groups, whereas GIT samples trended towards an increase in alpha diversity metrics for the MF group when visually compared with the HC and IF groups, but these trends were not significant (Fig. 2). There was also no significant difference in beta diversity between groups in either vagina or GIT samples (Figure S3, Table S7).

### Microbial trends in the vagina microbiome in treatment groups

Eight bacterial taxa were identified in all treatment groups: *L. crispatus*,* L. iners*,* L. jensenii*,* L. gasseri*,* Lactobacillus* spp. (consisting of > 70 unique ASVs), *L. acidophilus*,* L. reuteri* and *Gardnerella vaginalis*. Despite a lack of significance at the beta diversity level, subtle differences in the relative abundance of taxa were observed between the fluconazole groups (IF, MF) and the HC group. In the unmerged data set from the 16 S (bacterial) region from the vagina, *L. crispatus* displayed a higher average relative abundance in the IF group with 47.6% when compared to the MF (41.1%) or HC (25.5%) groups (Figure S3). *L. iners* also showed variability in average relative abundance between groups (MF 3.0%, IF 18.9%, and HC 8.1%). However, these differences were not significant.

In the IF and MF groups all participants that were categorised into CST-I (*n* = 8) displayed high average relative abundance of *L. crispatus (*> 90.0*%)*, whereas in the HC group this was the case for only one of the three of the participants with CST-I(98%) (Figure S3). Overall, IF group participants showed a higher average relative abundance of the *L. iners* dominated CST-III (94%) in comparison to other groups (HC 80%, MF 81%) (Figure S3).

Different bacterial and fungal taxa were observed in the VMB across all three participant treatment groups, but only two vaginal fungal species were observed consistently in all groups, they were: *Candida albicans* and *Malassezia globosa*. Moreover, *Saccharomyces cerevisiae* was not identified in the VMB of the MF group and *Rhodotorula mucilaginosa* was not identified in the HC group.

**Fig. 3:**
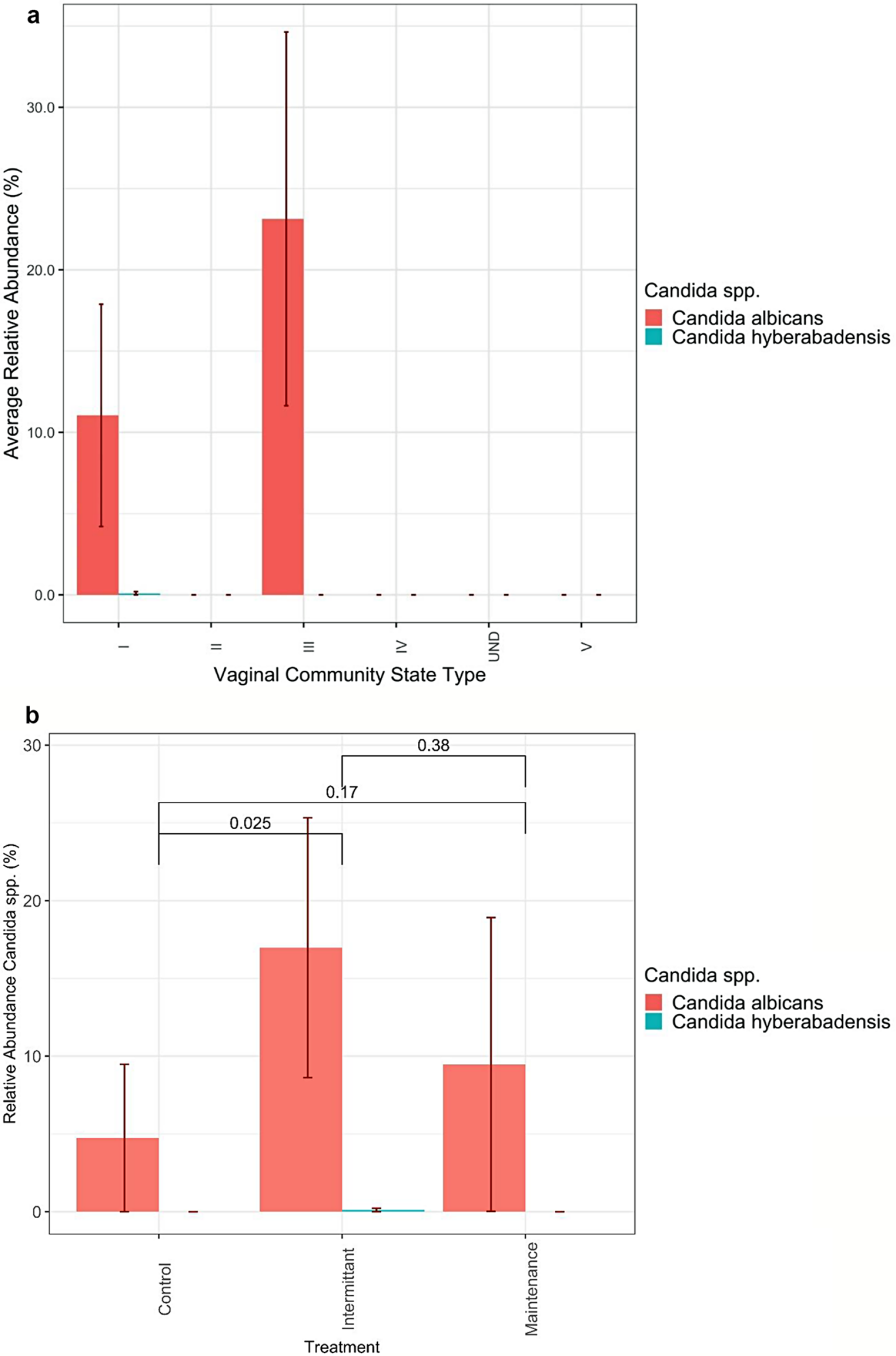
**a ***Candida* spp. relative abundances classified via CST group. Data displays relative abundance of *Candida* spp. in the vagina of participants depending on their Community State Type classification. Data were merged between ITS and 16 S sites from vaginal samples. Testing of the mean relative abundance differences between groups were conducted with Kruskal-Wallis one way analysis of variance. Data were rarefied prior to plotting. **b ***Candida* spp. relative abundances in the vagina. Data displays relative abundance of *Candida* spp. in the vagina of participants depending on their treatment group (IF, MF or HC). Data were merged between ITS and 16S sites from vaginal samples. Testing of the mean relative abundance differences between groups were conducted with Kruskal-Wallis one way analysis of variance. Data were rarefied prior to plotting

Overall, *Candida* spp. vaginal carriage was identified in nine participants HC (*n* = 1), IF (*n* = 6), MF (*n* = 2). All participants displaying vaginal *Candida* spp. belonged to either bacterial CST-I or CST-III (*n* = 8) (Fig. 3a). *Candida albicans* dominated among all *Candida* spp. in 95% of all *Candida* spp. positive samples in the vaginal environment. Other non- *albicans Candida* (NAC) species accounted for the remaining 5%.

A significant difference in *Candida* spp. VMB abundance was observed between the IF and HC groups (*p* = 0.025), with a greater average, yet non-significant, relative abundance in the IF and MF groups (*p* = 0.38) (Fig. 3b). *Candida albicans* was the most dominant *Candida* spp. observed. In interkingdom analysis four vaginal samples showed a higher relative abundance of *C. albicans* than their dominant CST associated *Lactobacillus* spp. (IF, *n* = 2, MF, *n* = 1, HC, *n* = 1, Fig. 1).

**Fig. 4:**
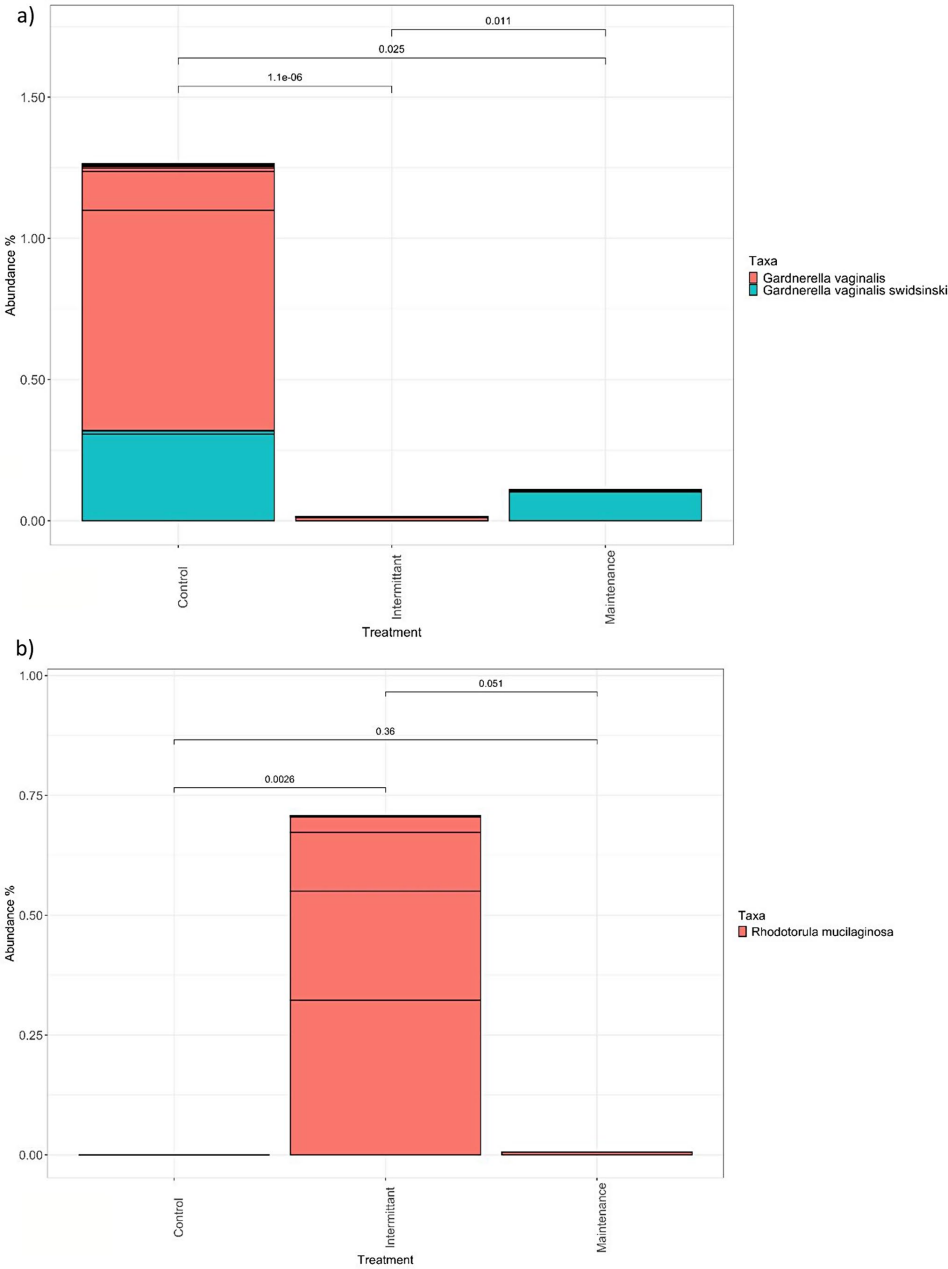
Effect of treatment on dominant species in the vaginal microbiome. Data displays relative abundance of selected relevant taxa in the vagina of participants grouped by treatment. Horizontal lines show different strains of the same species represented in the cohort **4a**) *G. vaginalis*, **4b**) *R. mucilanigosa*. Testing of the mean relative abundance differences between groups were conducted with Kruskal-Wallis one way analysis of variance. Data were rarefied prior to plotting

Specific bacterial and fungal species were assessed for interkingdom between-group differences. The between group difference of the vaginal bacterium *G. vaginalis* was significant, with an elevated abundance observed in the HC group compared to the IF and MF groups (Kruskal Wallis *p* < 0.05) (Fig. 4a). A significantly greater average relative abundance of vaginal *R. mucilanigosa* (microfungus) was observed in the IF group compared to both the HC and MF groups (*p* < 0.05) (Fig. 4b).

### *Candida albicans* in the gastrointestinal microbiome site

*Candida albicans* was also the dominant *Candida* spp. in the GIT environment (Table S6).

Compared to other groups, the IF group showed no significant dominance of C. *albicans* in the GIMB and the HC and MF groups had very low GIMB abundance of any *Candida* species (Table S6).

GIT samples were analysed for differences in the abundance of Firmicutes and Bacteroidetes and the MF group displayed a lower average relative abundance of Bacteroidetes compared with the HC and IF groups (Figure S5).

### Vaginal and GIT *Candida* pairing and dual site *Candida* carriage (co-carriage)

**Fig. 5:**
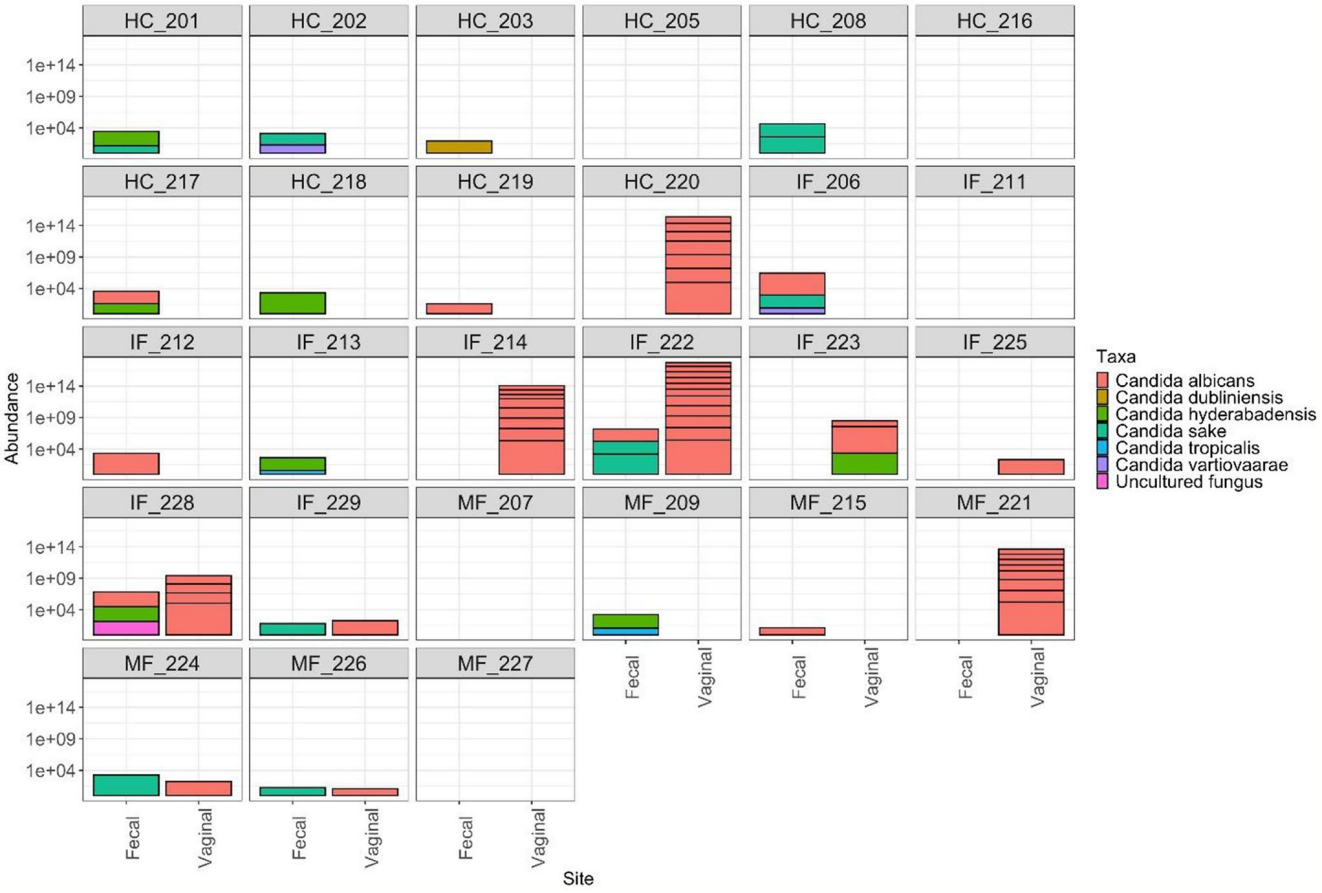
*Candida* carriage and pairing in gastrointestinal and vaginal microbiome. Data displays raw abundance of *Candida spp*. in the vagina and GIT shown per participant to demonstrate dual site relationships. Horizontal lines show a different strains of the same species. Data were normalised prior to plotting

Dual site carriage of *Candida*, where both the GIT and vagina sites have an occurrence of *Candida* spp., was assessed. Five participants (18.5%) of the overall sample recorded dual carriage of *Candida spp.* All of these samples originated from fluconazole users (IF = 3, MF = 2) (Fig. 5). *Candida* species were not consistently identified in both sites e.g., participants displayed GIT *Candida* spp. without any vaginal *Candida* spp. and vice versa (Fig. 5). Twelve participants (44.4%) displayed *Candida* spp. only in the GIT samples (HC = 7, IF = 3, MF = 2), only three (25%) of these were *C. albicans*. Five (18.5%) displayed no *Candida* spp. in either site (HC = 2, IF = 1, MF = 2) (Fig. 5).

When co-carriage was assessed for pairing e.g., the same species occurring in both the VMB and GIMB, only two participants in the IF group displayed a vaginal/GIT *C. albicans* pairing. When paired samples were assessed for *Candida* spp. vaginal abundance, abundance was higher. No NAC species were identified as being paired between sites (Fig. 5).

Two NAC species were seen predominately in the GIT environment; *C. saken* = 8 (HC = 3, IF = 3, MF = 2) with no vaginal carriage and *C. hyderabadenis*, which was predominantly found in the GIT *n* = 6 (HC = 3, IF = 2, MF = 1), none of these six displayed it vaginally. It was, however, observed in the vaginal sample for one IF participant (3.7%) (Fig. 5). Overall, five participants (18.5%) presented with *Candida spp.* only in the vagina (HC = 1, IF = 3, MF = 1).

### Vaginal and GIT *Lactobacillus* pairing and dual site *Lactobacillus* carriage (co-carriage)

Similar to assessing for *Candida* spp. co-carriage, the samples were also assessed for *Lactobacillus* spp. co-carriage and pairing. The majority of GIMB samples (*n* = 24 out of 27) did not display any *Lactobacillus* spp. at any site. Only three participants presented with co-carriage of *Lactobacillus* spp. (HC, *n* = 1, IF, *n* = 1, MF, *n* = 1). No pairing was observed between sites (Figure S6).

### Presence of Non- *Candida* fungal spp. in the Vagina

Fungal species that did not belong to the *Candida* genus were identified across groups in the VMB. 90% of the IF group (*n* = 9, 90%) presented with fungi including *Candida* and non-*Candida* species, compared to 72% of the MF and 50% of the HC groups (Table S5).

When dominant vaginal fungal characteristics were assessed, five IF participants (50%) showed dominance of non-*Candida* fungi. Four MF (57%) and four HC (40%) participants displayed non-*Candida* fungal kingdom dominance (Table S5). In the IF treatment group participants, #211 and #212 displayed *R. mucilaginosa* (average relative abundance 32.4% and 38.4%) with a CST-I bacterial microbiome (average relative abundance 63.0 & 61.1%). Participant #229 displayed *I. orientalis* dominance (16.2%) and a small < 1% abundance of *C. albicans* in an CST-III (83.5%) environment. Participant #223 displayed *C. albicans* dominance (9.5%) and five other non-*Candida* spp. were observed, with *M. globosa* (4.6%) in a CST-I (72.3%) environment being the most abundant species. Participant #226 in the MF group displayed a diverse mycobiome of 20 different species, 19 of which were non-*Candida* spp. *M. globosa* was the most abundant (2.0%), found with a CST-I (85.5%) bacterial community. The HC group participant #218 showed *S. cerevisiae* dominance (41.3%) with unassigned CST *L. acidophilus-*dominance. Two HC group participants displayed interkingdom relative abundance of fungal microbes higher than their dominant CST bacterial community; #220 was dominated by *C. albicans* (47.4%) and also carried five other non-*Candida* spp. at low levels and a CST-I (41.2%) bacterial environment. Participant #202 displayed *I. orientalis* (45.3%) as the dominant fungus and bacterial CST-IV with *G. vaginalis being* dominant (31.0%).

## Discussion

To our knowledge, this is the first study to compare the interkingdom VMB-GIMB of women with RVVC using oral fluconazole. It provides insight into interkingdom microbiome trends of typical RVVC patients and the impacts of recurrent treatments on dual-site microbiomes. The data adds to the discussion of *Candida* reservoirs’ in recurrency, and non-*Candida* fungal characteristics which may be linked to recurrency. It also prompts further evaluation of long-term therapy from a microbiome health perspective for both vaginal and gastrointestinal microbiomes.

### Fluconazole impacts on vaginal CST characteristics

Prior literature suggests that the RVVC vaginal bacterial and fungal microbiome are similar to that seen in healthy women when viewed at the phylum and genus level [31–33]. However, an intermediate disordered bacterial state with *L. iners* predominance has been observed post fluconazole [31, 34]. Overall, our study findings agree with the former, suggesting that the VMB of RVVC participants following differing fluconazole treatment regimes (IF and MF) are similar to non-RVVC women in microbial richness and diversity, defined by alpha diversity indices. We did not observe a predominance of a bacterial intermediate state such as *L. iners* dominated samples as part of bacteria-only analysis in the MF group, which would be more indicative of the recency of fluconazole treatment described in the literature [31]. However, the IF group did display a higher number of CST-III (*L. iners*) dominated samples. The consistency of maintenance dosing likely stabilises the bacterial VMB preventing shifts, which intermittent use does not provide. Whilst community compositional differences in richness and diversity in the interkingdom analysis between the fluconazole groups (IF and MF) and the HC group were not observed, the rarefied plot suggests that trends may become more significant in a larger population.

Consistent with prior research, study participants from all groups displaying vaginal *Candida* spp. belonged to either CST-I or CST-III [12]. This notable confirmatory finding highlights the predominance of *Lactobacillus* dominated microbiomes in people with RVVC using fluconazole with persistent vaginal *Candida* spp carriage. When viewed in isolation as unmerged bacterial data, the difference in *L. iners* relative abundance between fluconazole groups (MF = 3.0% vs. IF = 18.9%) and the higher amount of *Candida* spp. carriage in the IF group is consistent with previous research that observed an increase in *L. iners* in individuals reporting positive *Candida* culture, and history of RVVC for > 6 months [12]. The predominance of *L. iners* in this group also correlates with the above average vaginal pH recorded 5.0 (+/- 0.4 ) as *L. iners* fails to maintain environmental acidity and may be associated with Bacterial Vaginosis [35]. Half the participants in the IF group that carried vaginal *Candida* spp. were categorised as CST-III with a high of 100% relative abundance of *L. iners* (unmerged bacterial dataset). Moreover, the CST assessment of the predominance of the key *Lactobacillus* between the groups showed a higher relative abundance of *L. crispatus* in the IF group (47.6.0% ) and MF group (41.1%) compared to the HC group (25.5%). *L. crispatus* is a prevalent *Lactobacillus* species in the vaginal microbiome which has been shown to have antagonistic properties to *C. albicans* [12, 36]. Our results suggest fluconazole usage does not decrease *L. crispatus* levels, and over time and with frequent repetitive use like that seen in maintenance therapy, may increase the species relative abundance and minimise transitional shifts, resulting in high levels of *L. crispatus* while *C. albicans* abundance decreases. *L. crispatus* is a robust lactobacillus that, when established in high abundance, is not easily disturbed by environmental and physiological challenges [15]. This important observation may shed light on controversial “RVVC like” clinical presentations such as Cytolytic Vaginosis (CV), a *Lactobacillus* overgrowth disorder, which has been postulated to be related to antimicrobial use, including fluconazole [37, 38]. A high relative abundance (> 70%) of *L. crispatus* has been observed in CV when assessed *via* NGS [39]. As fluconazole use may be associated with higher relative abundance of *L. crispatus* and a reduction of *C. albicans*, the hypothesis that extended and frequent fluconazole therapy contributes to a CV presentation [38] seems to be substantiated by our results and requires further investigation.

### Fluconazole impacts on gastrointestinal microbiome

The results suggest that GIMB reservoirs of GIT *Candida* are unlikely to be involved in re-infection and recurrence cycles in RVVC, and that extended use of fluconazole may have unfavourable impacts on the GIMB.

NGS has not been used to assess RVVC gastrointestinal microbiome and fluconazole dosing relationships before [40]. In a murine study, Firmicutes were increased in the GIMB following fluconazole administration [40]. In this study, Firmicutes was the dominant fecal bacteria phylum observed in all groups. In the MF group the Firmicutes Bacteriodetes relationship, showed a lower ratio of Bacteroidetes to Firmicutes. This type of Firmicutes/Bacteriodetes ratio has been linked to GIMB dysbiosis, obesity, and metabolic disease [41] and requires further analysis. Suggesting a potential negative impact on GIT microbiome health from fluconazole maintenance therapy. However, the impacts of dietary and lifestyle changes, commonly adopted to gain control over RVVC symptoms, cannot be excluded [2].

### *Candida* burden: *Candida* abundance across groups and between sites

The *Candida* burden constitutes the relative abundance and number of *Candida* species in multi-sites and is significant for the pathophysiology of RVVC [16, 42, 43]. In this study we explored the relationship between fluconazole use and the abundance and carriage of *Candida* species at different sites. Consistent with the fungistatic mechanism of action of fluconazole a similarity between the MF and HC groups in their overall vaginal *Candida* carriage was observed. Likewise, the significantly larger distribution of *Candida* in the IF group compared to the HC group (*p* = 0.025) is consistent with prior literature where *Candida* presence post-therapy is associated with resistant traits and relapse [5, 12, 16]. Whilst we were unable to define if a relationship existed between the relative abundance of vaginal *Candida* spp. and an impending symptomatic episode, neither frequency of symptoms nor the subsequent fluconazole use in the IF group appeared to be associated with positive vaginal *C. albicans* carriage.

Whilst relationships were observed between CST and carriage, the connection to CST and relative abundance of *Candida* was varied in the IF group. As all participants were asymptomatic the persistence of vaginal *Candida* carriage and varying relative abundance in the MF group may potentially be indicative of *Candida* resistance traits and the protective traits of differing CST which may determine how likely they are to become symptomatic on cessation of treatment [16]. Further investigation and resistance testing would be advantageous to establish potential patterns of risk of relapse in all RVVC patients in particular people on MF who show persistent *Candida* carriage. As our study used a novel, merged interkingdom dataset analysis for assessing the VMB and GIMB, it highlighted the potential to apply a CST type categorisation in a larger cohort study, like the one used for vaginal bacterial species [28, 32]. This would allow further analysis and identification of patterns associated with the relative abundance relationships between fungal and bacterial kingdoms in health and in disease states like RVVC. In particular, the relative abundance of *Candida spp.* relationship to dominant Lactobacillus subtypes seen in the VMB analysis.

As a result of recurring infections, NAC species commonly colonise, and their resistance to antifungal agents like fluconazole may result in their persistence [44, 45]. This is mostly observed with *C. glabrata* as it is the second most implicated *Candida spp.*, after *C. albicans*, in VVC and RVVC [45]. We did not identify RVVC related NAC species such as *C. glabrata*, *C. krusei*, *C. dubliiniensis*, and *C. parapsilosis* [45] at any site for any participant. *C. tropicalis* was identified in the GIMB of one participant in the MF group but was not seen vaginally. The absence of NAC species vaginally in this cohort is likely reflective of the study requirement to be asymptomatic and responsive to fluconazole treatment [6]. Culture-based data which focus on *Candida* spp. suggests that 20% of a healthy population carries vaginal *Candida* species [12, 42, 46]. The HC group showed a 10% vaginal carriage of *Candida* spp. *via* NGS which may be considered low, as NGS studies have identified asymptomatic carriage can be as high as 33–98% [12, 42, 46]. This may be due to the overall selection criteria which excluded factors that could have impacted VMB such as IUD’s and progesterone only contraceptives [47, 48].

### Gastrointestinal re-infection reservoir of Candida

Whether the GIT serves as a reservoir for *Candida* species causing infection and re-infections in VVC and RVVC, remains unconfirmed due to a lack of specific studies [15, 16, 49]. The review of dual site carriage of *Candida* spp. and pairing of *Candida* spp. between sites showed inconsistent *Candida* identification between individuals in both sites. The pairing of species in both sites was also uncommon. In this study, the only two RVVC group samples that displayed pairing (IF group) had an interkingdom relative abundance of *C. albicans* greater than 34% in the VMB and < 1% in the GIMB. Thus, it seems unlikely that an intestinal reservoir of a specific *Candida* spp. is directly implicated in ongoing RVVC symptomatic episodes for RVVC patients. Rather, the vaginal environment and host conditions seem more important for the persistence of vaginal *Candida* infection than a large GIT reservoir. While GIT carriage of *Candida* could be associated with initial VVC infection pathogenesis, the absence of *Candida* pairing and only 20% *C. albicans* carriage in the HC GIMB suggests it is unlikely.

### Non-*Candida* fungal carriage

Microbially associated disorders are increasingly recognized as interkingdom processes, challenging a one pathogen, one disease perspective [50]. As studies to date have largely not explored the fungal VMB alongside bacterial VMB, a non-*Candida* fungal species-level interaction between bacterial and *Candida* spp., as well as the host response can’t be excluded and may be responsible for previously reported microbiome inconsistencies [46, 51]. The diversity of the fungal component of the VMB (70%), and non-*Candida* dominant species observed in the IF group, compared to 28% of MF and 30% of the HC group, requires further investigation in relation to fluconazole use and length of time on therapy, as undefined, possibly complex relationships between non-*Candida* fungal microbes and *Candida* spp. may contribute to its persistence and infection opportunity. It is reported that a small number of mycoses colonise asymptomatically, including *Candida* spp., and *Malassezia* spp., both observed in our data across all groups in an asymptomatic state [52]. Furthermore, the significance of *R. mucilaginosa* as the dominant fungal species in IF participants is of interest, as it has been implicated in infections at other body sites and has previously been linked to vaginitis [53]. It is documented that non- *Candida* fungal species persist and can acquire transient antifungal resistance to antifungals like fluconazole [54]. Specifically, for *R. mucilaginosa* pathogenic traits have been observed when the host is immunocompromised or the host environment is disturbed by antimicrobial treatment [53].

The diversity of the fungal VMB was also considered in relation to fluconazole use. In this study several participants displayed more than one non-*Candida* fungal species. Participant #226 (MF) had a diverse mycobiome with 19 non-*Candida* spp. and a < 0.1% of *C. albicans*. This participant had been on a form of digressive maintenance therapy for 18 months with the most current regime consisting of 150 mg fluconazole every 2 weeks. This was in comparison to the other MF users who had used maintenance therapy for between 1 and 5 months and did not display diverse mycobiomes.

Whilst this study’s sample size is insufficient to conclude, the potential for long-term fluconazole treatment to increase fungal species diversity and enhance resistant species growth is of interest. As such changes in mycobiome diversity over time due to fluconazole use requires further research so that non-*Candida* fungal selection and growth impacts associated with fluconazole’s long-term use can be better understood [54].

### Strengths

To our knowledge, this is the first study to compare the interkingdom VMB-GIMB of women with RVVC using oral fluconazole. Utilising an exploratory novel interkingdom approach to analysis is a strength of this study, as it provides insight into the fungal and bacterial microbial relationships at dual sites. It allowed the visualisation of vaginal bacterial data as unmerged data allowed to draw correlations between prior literature and our findings. Sampling was timed to occur in the mid-luteal phase, representing a higher risk time for RVVC patients for symptomatic recurrence. The luteal phase is however reported to be more stable in terms of microbial composition which correlates with the higher circulating concentrations of sex hormones such as estrogen and progesterone [55], and therefore allows a between-group comparison without potential hormonal-induced VMB fluctuations associated with cycle stage. All participants sampled had no active symptoms and thus the reported microbiome characteristics were indicative of relative health for the individual at baseline in an asymptomatic state. As such the study provides true insights into potential characteristics that could put women with RVVC at risk of a symptomatic flare and be reflective of the impacts of fluconazole.

### Limitations

As an exploratory investigation, not all data represented significant results due to low participant numbers and as such emergent trends could not be confirmed. More conclusive observations would be possible through a larger-scale exploration, and ethnicity variances in the population would also be better represented as a factor related to CST classification tendencies [28]. In addition, longitudinal microbiome analysis and hormonal assessment of this population could provide an understanding of the interkingdom and cyclical fluctuations associated with symptoms and their resolution. Exploration of these microbiome sites with multiple sample points in a single month over a number of months could enable the identification of more significant trends associated with both RVVC and fluconazole use. This approach would also reduce difficulties encountered when assessing species that were most different amongst the treatment groups.

The sample collection for some remote participants resulted in a delay between sample collection and freezing at -80 C (up to 24 h). The Copan FloQ swab is validated for swabbing with immediate freezing with constant vaginal microbiome accuracy [56]. Covid-19 associated changes to methodology necessitated a variation to sample receipt. No stabiliser was used before shipping the samples. It is possible that some DNA degradation took place in the samples, as no published Copan FloQ vaginal swab stability studies with freezing delay are available to date. However, internal validation of DNA stability on a dry Copan FloQ vaginal swab has been confirmed by Invivo Healthcare labs in the UK (S. Forrest personal communication October 26, 2024). In future larger studies utilising these swabs a stabiliser could be considered. All samples yielded results.

An in-depth analysis of the GIT data is not reported as it focusses on the relationship between microbiome sites, emphasising *Candida* carriage and pairing data. Future analysis of the GIMB data may add information important for understanding the impacts of fluconazole, as users have reported gastrointestinal function changes such as constipation, which may be linked to the trends we observed in phylum and diversity change.

It was expected that some individuals may not have detectable levels of vaginal fungi due to read counts and thresholds as Fungal ITS1 sequencing has previously been reported to produce lower overall reads [43]. Concurrent measurement of culture and quantification of the amount of *Candida* present *via* qPCR would be advantageous to understand differences in *Candida* load between groups and may contribute to disease pathology, indicative of symptomatic recurrence onset [12].

## Conclusion

This research significantly contributes to the existing body of knowledge in RVVC and initiates an important discussion about the potential impacts of oral fluconazole therapy on the VMB and GIMB. We found that the VMB and GIMB of people using fluconazole do not differ in diversity metrics compared to healthy controls, however relative abundance of *C. albicans* vaginally was significantly higher between the IF and HC groups highlighting a difference in *Candida* carriage and symptomatic tendencies. Likewise, trends in CST associated Lactobacillus and higher relative abundance in fluconazole users’ needs to be explored further. The impact of fluconazole requires further analysis to establish long-term efficacy and safety regarding microbiome health, as a potential selective survival and loss of bacterial and fungal species could be implicated in symptomatic recurrence of RVVC, but also in the development of other vaginal conditions.

This study also provided insights into the role of fecal *Candida* reservoirs in recurrent symptomatology suggesting that it is an unlikely cause of symptomatic relapse. The GIMB phylum level differences in the MF group highlights the need to explore the impacts of long-term use of fluconazole. Notably, questioning and on monitoring of GIT functional change in patients on extended fluconazole which may be reflective of impacts on GIT health. As fluconazole users in this study represent a typical RVVC population, the examination of both microbiome sites adds to the current understanding of microbial trends in the condition. Further, larger cohort exploration of these microbiome sites could help identify newer patient specific interventions and diagnostics based on trends in diversity seen in both the VMB and GIMB and inform safety data associated with microbiome guardianship.

## Electronic supplementary material

Below is the link to the electronic supplementary material.

Supplementary Material 1: The following are included as supplemental materials: Table S1. Fluconazole Use Characteristics. Table S2. Sexual Behaviour Variables. Table S3. Subject Variables. Table S4. Sequence Read Details (TO LINK HERE). Table S5. Vaginal Taxa. Table S6. Fecal Taxa. Table S7. Beta Diversity Testing.

## Data Availability

Sequence data are available on the National Center for Biotechnology Information (NCBI) Sequence Read Archive under BioProject accession number SUB14294064.
