# Supplementary material for: The impact of fluconazole use on the fungal and bacterial microbiomes in recurrent Vulvovaginal Candidiasis (RVVC): a pilot study of vaginal and gastrointestinal site interplay
Source: Eur J Clin Microbiol Infect Dis. 2024 Nov 26;44(2):285–301. doi: 10.1007/s10096-024-04999-1 (PMC11754358; doi:10.1007/s10096-024-04999-1)
Supplement: Supplementary file 1 — Supplementary Material 1: The following are included as supplemental materials: Table S1. Fluconazole Use Characteristics. Table S2. Sexual Behaviour Variables. Table S3. Subject Variables. Table S4. Sequence Read Details (TO LINK HERE). Table S5. Vaginal Taxa. Table S6. Fecal Taxa. Table S7. Beta Diversity Testing. [file 10096_2024_4999_MOESM1_ESM.docx]

**Supplementary Materials**

**Table S1. Fluconazole and antifungal drug use characteristics of IF and MF groups.** *Data are expressed as mean, SD,* n/N (%)

| **Variable** | **IF**  **(n=10)** | **MF**  **(n=7)** | **P Value** |
| --- | --- | --- | --- |
| **Fluconazole use in the 14 days preceding sample day** | 0(0.0%) | 7(100.0%) | (p=<0.001) |
| **Maintenance fluconazole therapy use**  ***Current***  ***In past*** | 0(0.0%)  3(30.0%) | 7(100.0%)  7(100.0%) | (p=<0.001)  (p=0.01) |
| **Mean time on current FM therapy (months)** | NA | 4±6.22 | NA |
| **Mean number of times use IF in the previous 12 months per person** | 6.0±2.8 | 2.0±3.8 | (p=0.02) |
| **Fluconazole as prescribed***  ***50mg daily***  ***50mg 1 x week***  ***50mg 3 x week***  ***150mg 1 x week***  ***150mg every 2 weeks*** | 0(0.0%)  0(0.0%)  1(10.0%)  9(90.0%)  0(0.0%) | 4(57.1%)  0(0.0%)  0(0.0%)  2(38.6%)  1(14.3%) | (p=0.006) |
| **Oral itraconazole use in the previous 12 months** | 3(30.0%) | 1(14.3%) | (p=0.60) |
| **Oral nystatin use in the previous 12 months** | 0(0.0%) | 1(14.3%) | (p=0.41) |
| **Prescribed extended vaginal clotrimazole therapy in the previous 12 months** | 1(10.0%) | 0(0.0%) | (p=0.00) |
| **Vaginal Boric acid use in the previous 12 months** | 4(40.0%) | 1(14.3%) | (p=0.34) |
| **Vaginal nystatin use in the previous 12 months** | 0(0.0%) | 1(14.3%) | (p=0.41) |

*as prescribed refers to the frequency of use as suggested by the health professional. Those in the IF group used the fluconazole dose as needed and in response to symptoms. Those in the MF group used fluconazole ongoing as part of symptom control

**Table S2. Sexual interaction characteristics of *HC, IF and MF groups in the 14 days prior* to sample day.*** *Data are expressed as mean, SD,* n/N (%)

| **Variable** | **Total**  **(n=27)** | \| **HC**  **(n=10)** \|  \| \| --- \| --- \| | **IF**  **(n=10)** | **MF**  **(n=7)** | **p Value** |
| --- | --- | --- | --- | --- | --- | --- | --- |
| **Engaged in sexual intercourse** | 21(77.7%) | 8(80.0%) | 8(80.0%) | 5(71.4%) | (p=1.00) |
| **Engaged in unprotected sex^a^** | 16(59.3%) | 5(50.0%) | 6(60.0%) | 5(71.4%) | (p=0.88) |
| **Used vaginal lubricant** | 11(40.7%) | 4(40.0%) | 5(50.0%) | 2(28.6%) | (p=0.88) |
| **Engaged in oral sex (to vagina)** | 16(59.3%) | 3(30.0%) | 8(80.0%) | 5(71.4%) | (p=0.09) |
| **Experienced post-coital vaginal symptoms** | 7(25.9%) | 2(20.0%) | 4(40.0%) | 1(14.3%) | (p=0.55) |

*Not in the 48 hours before sampling

^a^Vaginal sex without male condom, vaginal dam, or internal condom

**Table S3. Subject variables of *HC, IF and MF groups.*** *Data are expressed as mean, SD,* n/N (%)

| **Variable** | **Total (n=27)** | \| **HC**  **(n=10)** \|  \| \| --- \| --- \| | **IF**  **(n=10)** | **MF**  **(n=7)** | **p Value** |
| --- | --- | --- | --- | --- | --- | --- | --- |
| **Vaginal pH ,  mean(SD)** | 5.0±0.4 | 5.1±0.4 | 4.9±.04 | 5.1±0.4 | (p=0.49) |
| **Sobel Score>3,  n(%)** | 0(0.0%) | 0(0.0%) | 0(0.0%) | 0(0.0%) | (p=1.00) |
| **Menstrual cycle or OCP equiv. day mean(SD)** | 20.48±2.7 | 21.2±1.5 | 19±3.7 | 21.6±1.8 | (p=0.09) |
| **Vulval washing frequency in last 14 days,  mean(SD)** | 16.18±7.6 | 12.9±6.2 | 17.5±7.6 | 19.0±8.8 | (p=0.22) |
| **Use of sanitary item in last 14 days,  n(%)** | 3(11.1%) | 2(20%) | 0(0.0%) | 1(14.3%) | (p=0.45) |
| **Used feminine wash, wipe, or spray in last 14 days,  n(%)** | 2(7.4%) | 1(10%) | 1(10.0%) | 0(0.0%) | (p=1.00) |

**Table S4. Sequence Read Details -Online data link will be supplied upon acceptance**

**Table S5. Vaginal Taxa -Online data link will be supplied upon acceptance**

**Table S6. Fecal Taxa -Online data link will be supplied upon acceptance**

**Table S7. Beta Diversity Testing.** Measuring statistical significance of beta diversity difference between the treatment group (MF, IF and HC) in the Vaginal and Fecal environment using Permutational Multivariate Analysis of Variance (PERMANOVA). Bray Curtis dissimilarity matrices were used. Data were rarified prior to plotting.

| Dataset | Grouping variable | DF | Sum of Squares | R^2 | F | P-value |
| --- | --- | --- | --- | --- | --- | --- |
| Vaginal samples | Treatment group | 2 | 0.7913 |  | 0.9902 | 0.4361 |
| Fecal samples | Treatment group | 2 | 0.7058 | 0.0842 | 1.1033 | 0.1896 |


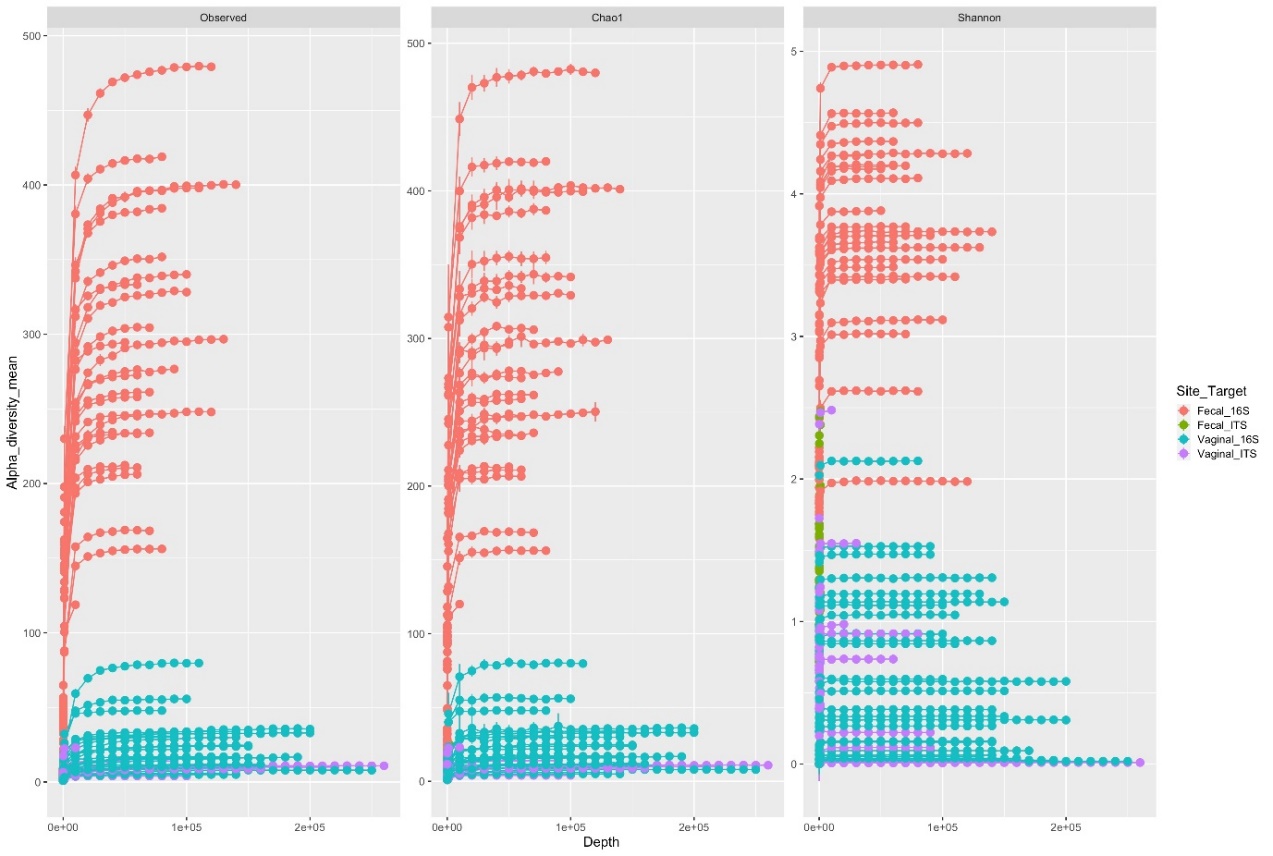


**Figure S1. Rarefaction curves of samples and sequencing depth.** Rarefaction curves indicate the impact of sampling depth on species observation in all samples. Individuals from three treatment cohorts: control (HC), RVVC patient with intermittent fluconazole use (IF) and RVVC patient with maintenance fluconazole use (MF).

*
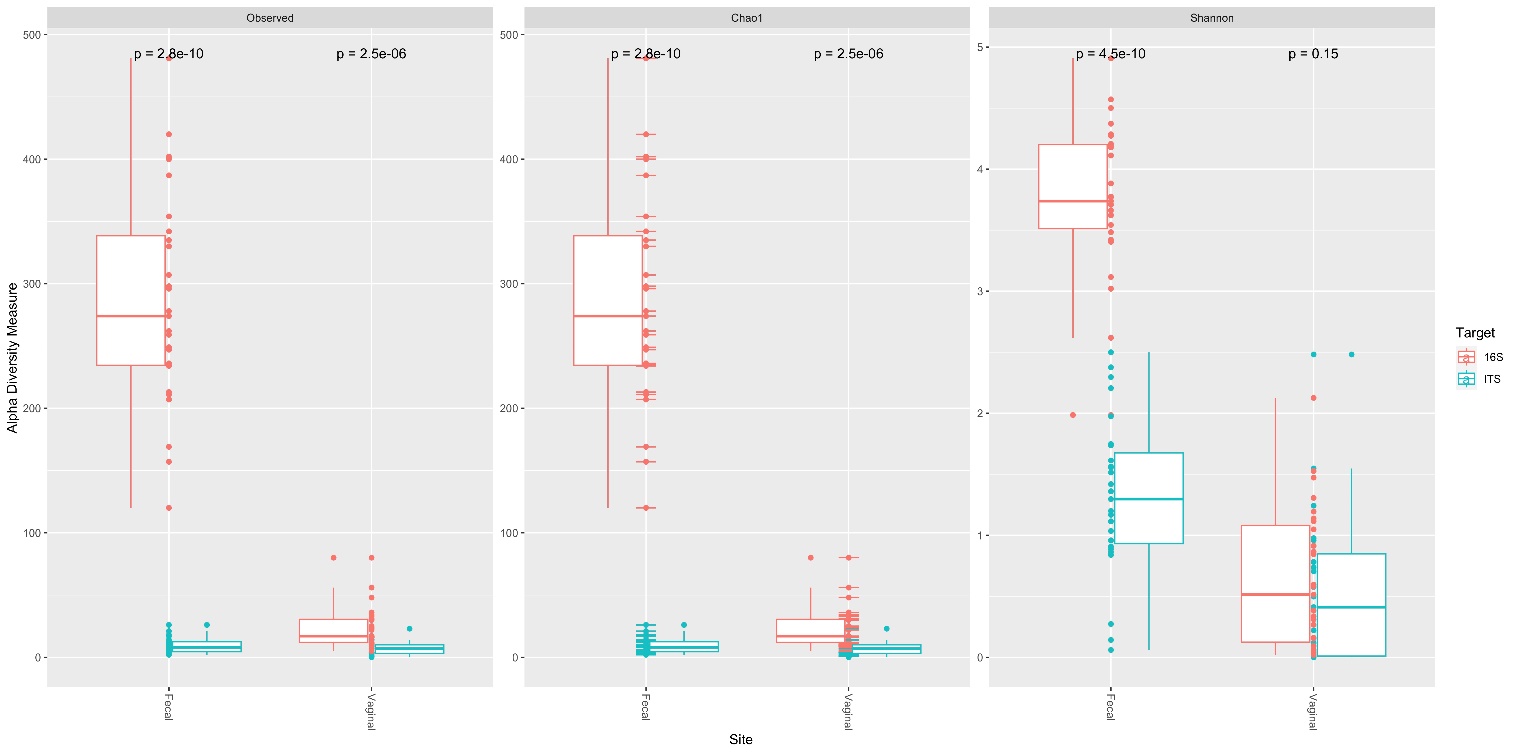
*

S2a)

*
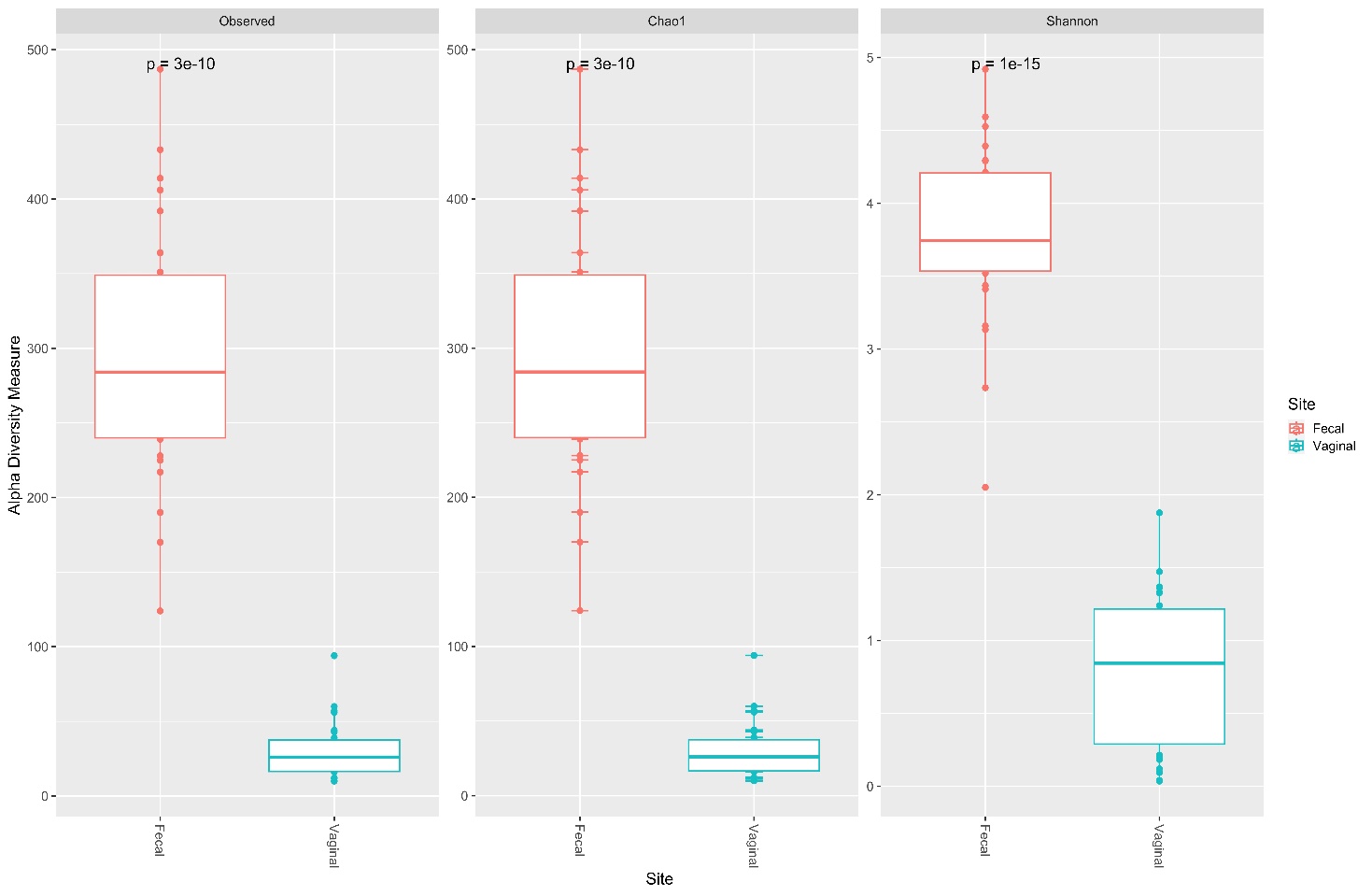
*

S2b)

**Figure S2. Alpha Diversity of fungal and bacterial microbiome in vagina and GIT with treatment for RVVC.** Alpha diversity in treatment groups for vagina and GIT indicating testing of mean between groups by Kruskal-Wallis. S2a) unmerged data, S2b)merged data


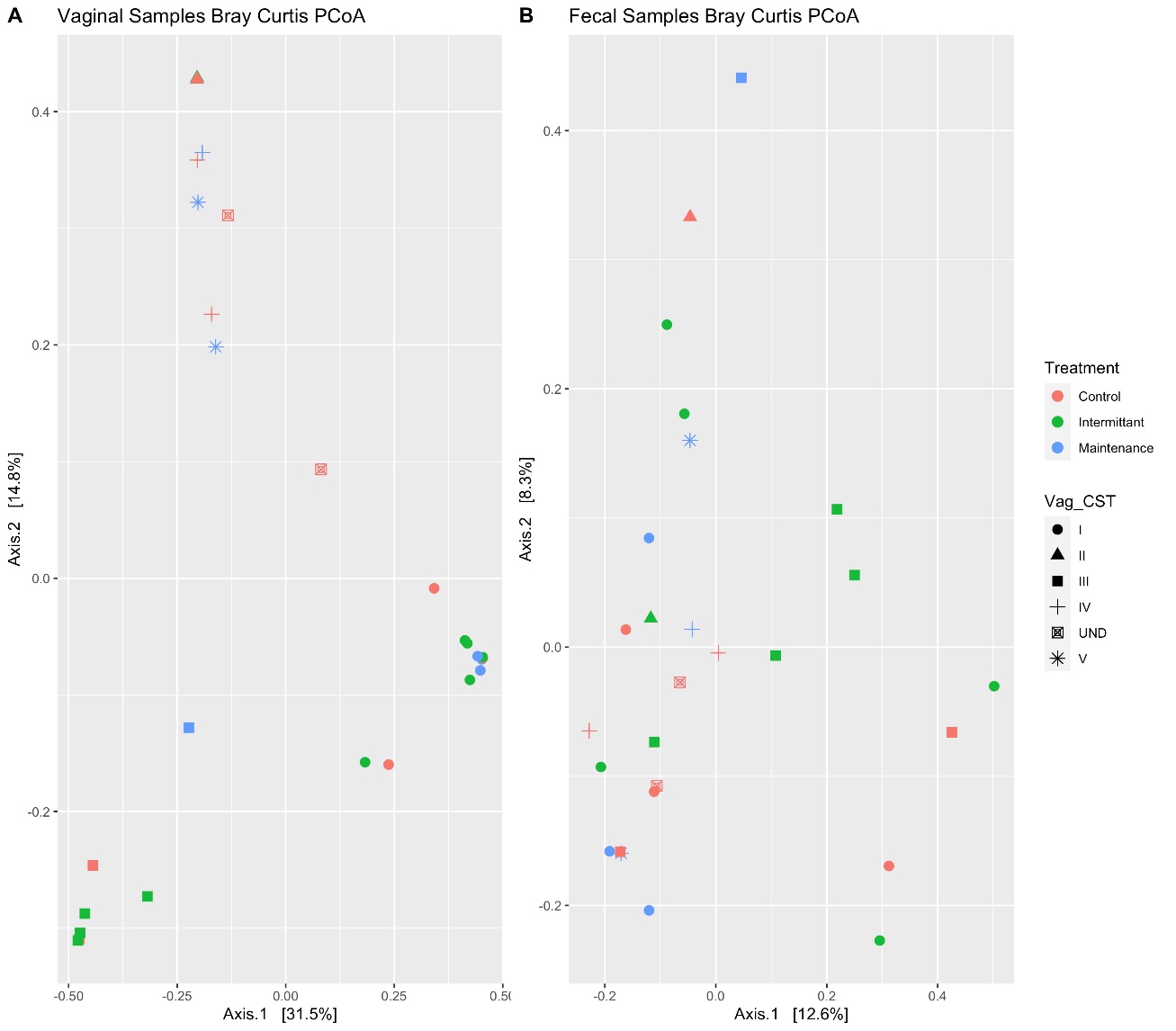


**Figure S3. PCoA plot of bacterial and fungal microbiome profiles in the vagina and GIT of participants.** Plot displays the microbiome based on Bray-Curtis dissimilarity matrix for individual samples from vagina (A) and GIT (B), coloured by treatment type and symbols shaped by community state type. Data were rarefied prior to plotting.


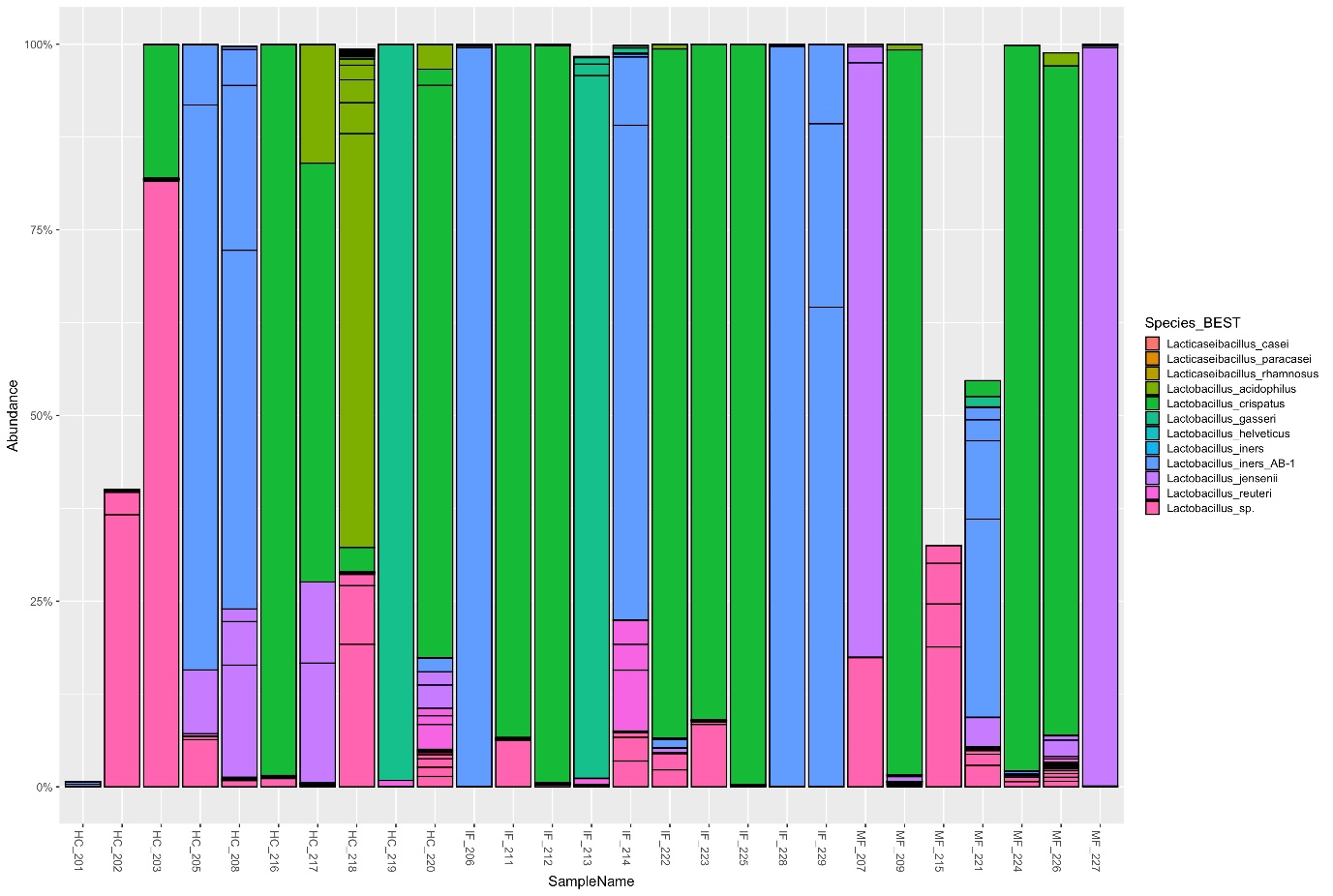


**Figure S4. Relative abundance of *Lactobacillus* spp. in the vagina of study participants.** Bar chart displays the relative abundance from rarefied data from 16S only (unmerged) reads in the vagina of study participants. Data were rarefied prior to plotting and Community State Type was determined from the dominant *Lactobacillus* spp. observed in each participant. Table 3 details the representative from CST’s in treatment groupings.


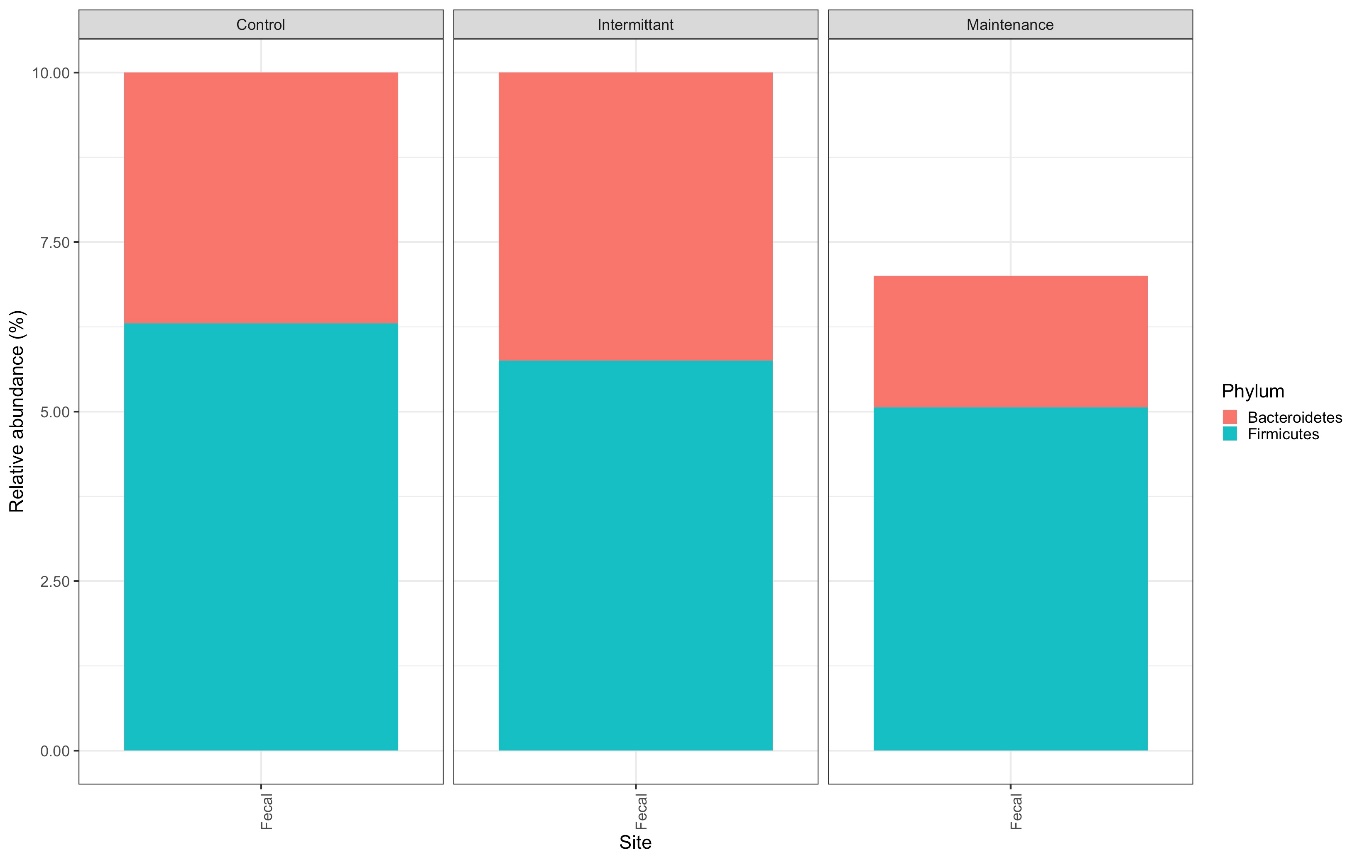


**Figure S5. Effect of treatment on dominant phylum in the gastrointestinal microbiome.**

Data displays relative abundance of Firmicutes and Bacteriodetes phylum in the GIT of participants grouped by treatment grouping. Data were rarefied prior to plotting.


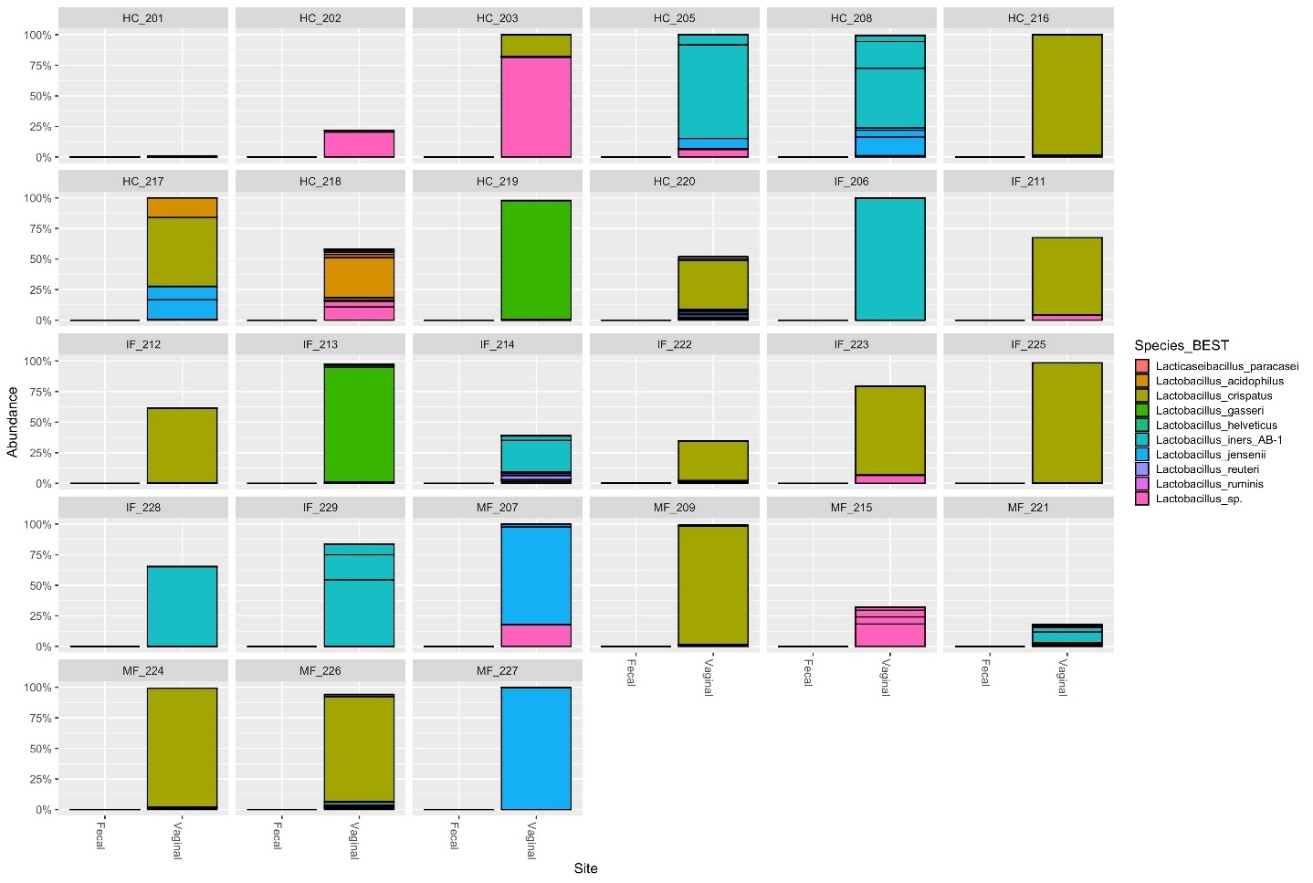
**Figure S6. *Lactobacillus* carriage and pairing in gastrointestinal and vaginal microbiome** Data displays relative abundance of *Lactobacillus spp*. in the vagina and GIT shown per participant to demonstrate dual site relationships. Data were rarefied prior to plotting.
